# Gender Related Differences in Kidney Injury Induced by Mercury

**DOI:** 10.3390/ijms130810523

**Published:** 2012-08-22

**Authors:** María H. Hazelhoff, Romina P. Bulacio, Adriana M. Torres

**Affiliations:** Pharmacology, Faculty of Biochemist and Pharmaceutical Sciences, National University of Rosario, CONICET, Rosario 2000, Argentina; E-Mails: mariaherminiahazelhoff@yahoo.com.ar (M.H.H.); robulacio@hotmail.com (R.P.B.)

**Keywords:** sex differences, kidneys, organic anion transporters, Oat1, Oat3, acute renal failure, mercuric chloride

## Abstract

The aim of this study was to determine if there are sex-related differences in the acute kidney injury induced by HgCl_2_ since female rats express lower levels of renal Oat1 and Oat3 (transporters involved in renal uptake of mercury) as compared with males. Control males and females and Hg-treated male and female Wistar rats were employed. Animals were treated with HgCl_2_ (4 mg/kg body weight (b.w.), intraperitoneal (i.p.)) 18 h before the experiments. HgCl_2_ induced renal impairment both in male and female rats. However, female rats showed a lower renal impairment than male rats. The observed increase in kidney weight/body weight ratio seen in male and female rats following HgCl_2_ treatment was less in the female rats. Urine volume and creatinine clearance decreased and Oat5 urinary excretion increased in both males and females, but to a lesser degree in the latter. Urinary alkaline phosphatase (AP) activity and histological parameters were modified in male but not in female rats after HgCl_2_ administration. These results indicate that the lower Oat1 and Oat3 expression in the kidney of females restricts Hg uptake into renal cells protecting them from this metal toxicity. These gender differences in renal injury induced by mercury are striking and also indicate that Oat1 and Oat3 are among the main transporters responsible for HgCl_2_-induced renal injury.

## 1. Introduction

The health effects of mercury are highly dependent on the different chemical forms of mercury. Dental amalgam is the major source of mercury vapor exposure in the general population and an association between the number of amalgam fillings and the concentrations of inorganic mercury in blood and urine has been reported [1,2]. Low concentrations of inorganic mercury are ingested in the diet, mainly as Hg^2+^. Occupational exposure occurs mainly in the chloralkali industries and in the gold mining industries [1–3]. High levels of mercury exposure may occur via the use of skin lightening beauty creams and soaps [3] and herbal drugs [4].

Sex-related differences in mercury handling in both animals and humans have been described. In terms of inorganic mercury, Hultman and Nielsen [5] reported significantly greater whole-body mercury retention as well as greater mercury accumulation in kidneys and spleens of male compared with female mice of several strains during prolonged exposure to mercuric chloride. In human studies, women were reported to have significantly higher urinary mercury concentrations than men with comparable numbers of dental amalgam fillings [6]. Studies on the excretion of organic and inorganic mercury in methylmercury-treated rats [7] showed faster whole-body clearance of mercury in females than in males. Similarly, studies on methylmercury exposure in human infants and children [8,9] as well as in animals [10,11] reported greater developmental effects in males than in females, consistent with higher overall mercury retention and lower rates of mercury excretion by males. Although numerous factors that might differentially affect mercury disposition have been reported [12], the biological mechanisms underlying sex-related differences in mercury excretion rates or susceptibility to mercury toxicity remain to be identified.

Inorganic mercury has a non-uniform distribution after absorption, accumulating mainly in the kidneys [13], causing acute renal failure [14]. Renal proximal tubular cells represent the primary target site where highly reactive mercuric ions rapidly accumulate and induce cell injury by binding to and inhibiting various sulfhydryl-containing enzymes [15]. Complex factors may account for this susceptibility, including the selective presence of specific transport systems involved in the uptake of mercuric ions by particular cell populations [16]. The extraction of mercury from kidneys does not appear to involve glomerular filtration but rather a direct secretory process whereby mercury moves from peritubular blood into the tubular lumen [17]. Hg^2+^ gains access to proximal tubular cells primarily via amino acid transporters in the luminal plasma membrane and the organic anion transporters 1 and 3 (Oat1 and Oat3) in the basolateral plasma membrane [15,18]. Bridges *et al.* [19] have recently demonstrated that multidrug resistance-associated protein 2 (Mrp2) plays an important role in the renal cellular elimination and secretion of Hg^2+^ in rats.

We have recently demonstrated that most of the renal injury (both histologically and biochemically as measured by blood urea nitrogen and creatinine levels) was abolished following HgCl_2_ treatment of *Oat1* knock-out mice. Thus, acute kidney injury by HgCl_2_ was found to be mediated mainly by Oat1 [20].

It has been described that gender differences in the rat renal cortical Oat1 and Oat3 appear after puberty and are determined by both a stimulatory effect of androgens (and progesterone in the case of Oat1) and an inhibitory effect of estrogens [21–23].

The aim of this study was to determine if there are sex-related differences in the acute kidney injury induced by HgCl_2_ since female rats express lower levels of renal Oat1 and Oat3 as compared with males.

## 2. Results and Discussion

**Figure 1:**
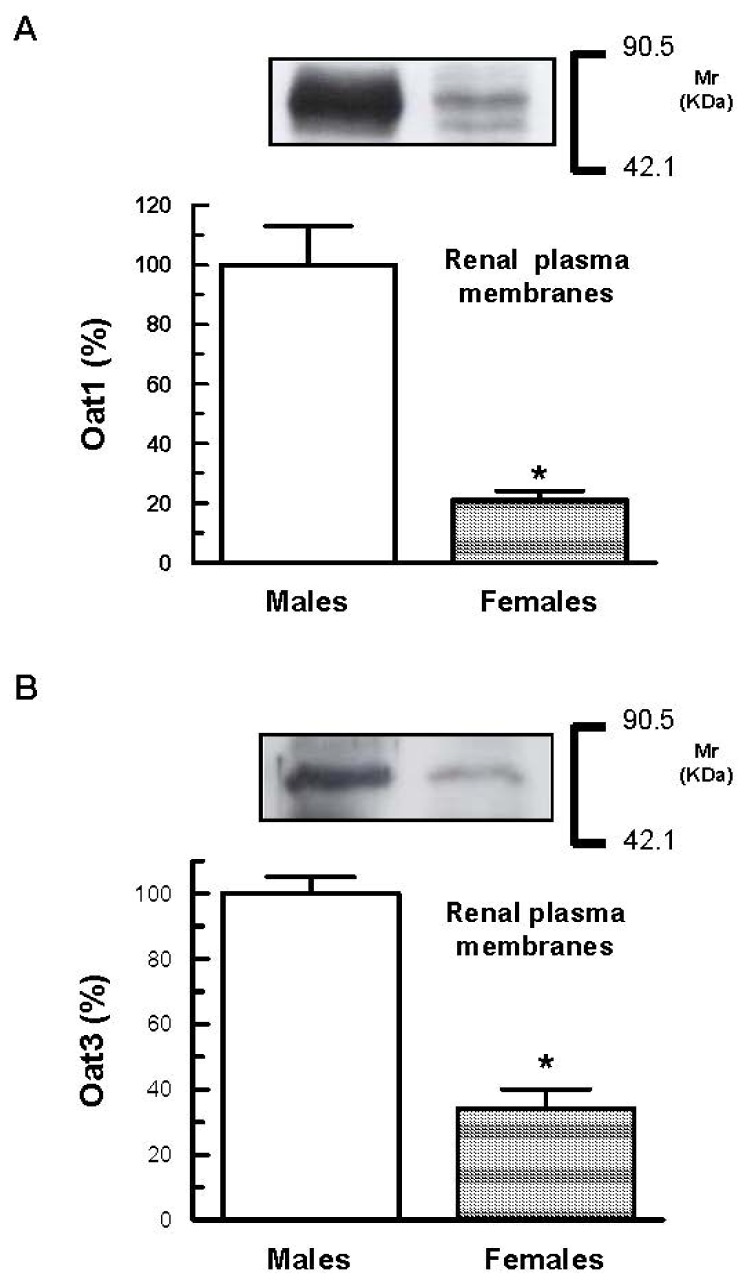
Western blotting for Oat1 (**A**) and for Oat3 (**B**) in plasma membranes (18 μg proteins) from kidneys of male and female rats. Proteins were separated by SDS-PAGE and blotted onto nitrocellulose membranes. The mean of the male levels were set as 100%. Each column represents the mean ± SEM from experiments carried out in four animals for each experimental group. * *p* < 0.05. Kaleidoscope Prestained Standards of molecular mass corresponding to bovine serum albumin (90.5 kDa) and to carbonic anhydrase (42.1 kDa) are indicated in the right of the figure.

As shown in Figure 1, in renal plasma membranes isolated from kidneys, male rats exhibited about 5-times and about 3-times higher densities of the Oat1 and Oat3-related protein bands, respectively, than did females, as previously described [22,23].

Table 1 shows the effect of HgCl_2_ treatment on the ratio of kidney weight to body weight, urinary volume, creatinine clearance and the urinary activity of alkaline phosphatase (AP) in male and female rats. The differences observed in these variables between control females and control males have been previously described in our laboratory and by other authors [24–26].

**Figure 2:**
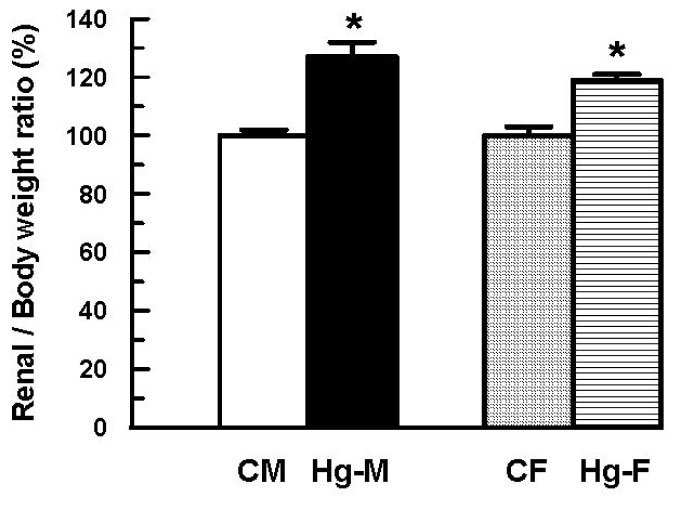
Effect of HgCl_2_ (4 mg/kg of body weight, i.p.) on the ratio of kidney weight to body weight in male and female rats 18 h after treatment. Each column represents the mean ± SEM from experiments carried out in four animals for each experimental group. * *p* < 0.05 *vs*. respective control. The mean of both control male and female levels were set as 100%. **CM**: Control Males; **Hg-M**: Hg-treated Males; **CF**: Control Females; **Hg-F**: Hg-treated Females.

**Figure 3:**
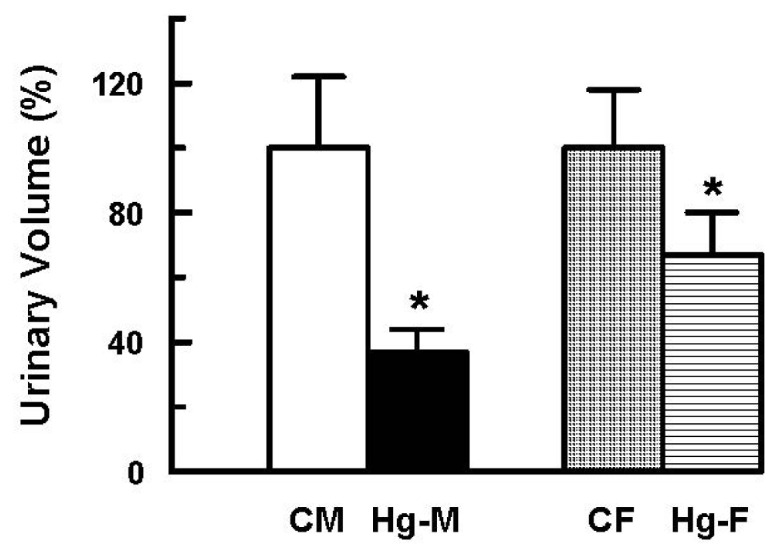
Effect of HgCl_2_ (4 mg/kg of body weight, i.p.) on the urinary volume in male and female rats 18 h after treatment. Each column represents the mean ± SEM from experiments carried out in four animals for each experimental group. * *p* < 0.05 *vs*. respective control. The mean of both control male and female levels were set as 100%. **CM**: Control Males; **Hg-M**: Hg-treated Males; **CF**: Control Females; **Hg-F**: Hg-treated Females.

**Figure 4:**
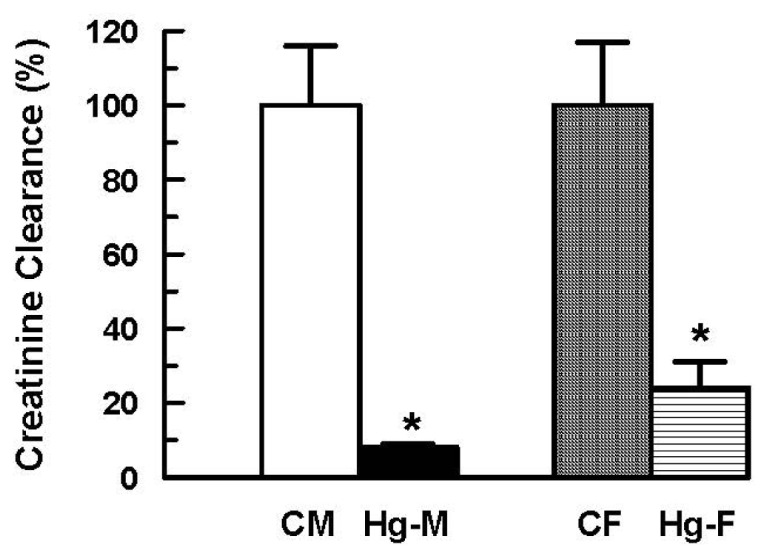
Effect of HgCl_2_ (4 mg/kg of body weight, i.p.) on the creatinine clearance in male and female rats 18 h after treatment. Each column represents the mean ± SEM from experiments carried out in four animals for each experimental group. * *p* < 0.05 *vs*. respective control. The mean of both control male and female levels were set as 100%. **CM**: Control Males; **Hg-M**: Hg-treated Males; **CF**: Control Females; **Hg-F**: Hg-treated Females.

**Figure 5:**
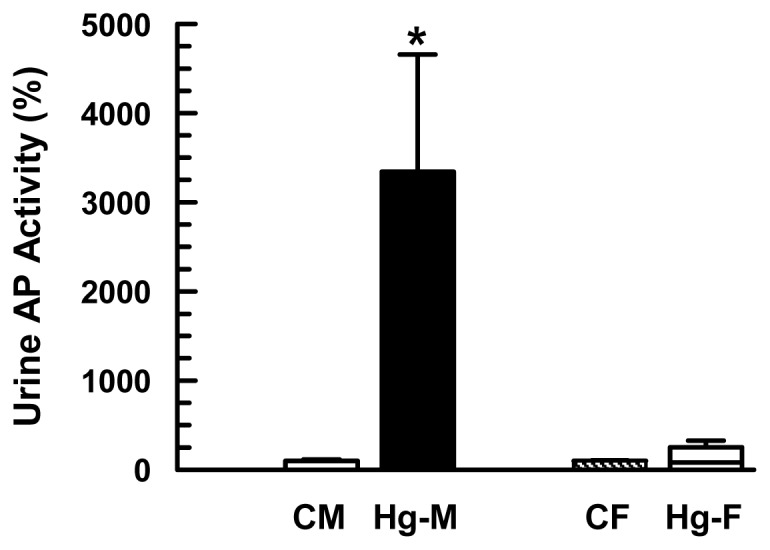
Effect of HgCl_2_ (4 mg/kg of body weight, i.p.) on the urinary alkaline phosphatase (AP) activity in male and female rats at 18 h after treatment. Each column represents the mean ± SEM from experiments carried out in four animals for each experimental group. * *p* < 0.05 *vs*. respective control. The mean of both control male and female levels were set as 100%. **CM**: Control Males; **Hg-M**: Hg-treated Males; **CF**: Control Females; **Hg-F**: Hg-treated Females.

The same parameters displayed in Table 1 are shown in Figures 2–5; expressed as a percentage in order to better appreciate the nephrotoxic effect of mercuric treatment for each sex.

Treatment of male rats with 4 mg of HgCl_2_/kg of body weight (i.p.) led to a significant increase in the ratio of kidney weight to body weight (Figure 2). To obtain other evidence regarding the role of Oat1 and Oat3 in the renal injury induced by mercury, female rats were subjected to treatment with 4 mg of HgCl_2_/kg of body weight. Following treatment with HgCl_2_, there was an increase in the ratio of kidney weight to body weight in female rats compared with vehicle-treated females. However, this increase was smaller than that observed in HgCl_2_-treated male rats (Figure 2).

In addition, significant decreases in urinary volume and in creatinine clearance were observed in male and female rats following HgCl_2_ treatment (Figures 3 and 4). However, the decreases in HgCl_2_-treated females were much smaller than those observed in HgCl_2_-treated male rats.

The urinary activity of AP has been widely used as a biomarker for renal injury [27,28]. Thus, the urinary activity of AP was measured, related to urinary creatinine concentrations. As shown in Figure 5, the activity of AP was only significantly increased in urine samples from HgCl_2_-treated male rats.

**Figure 6:**
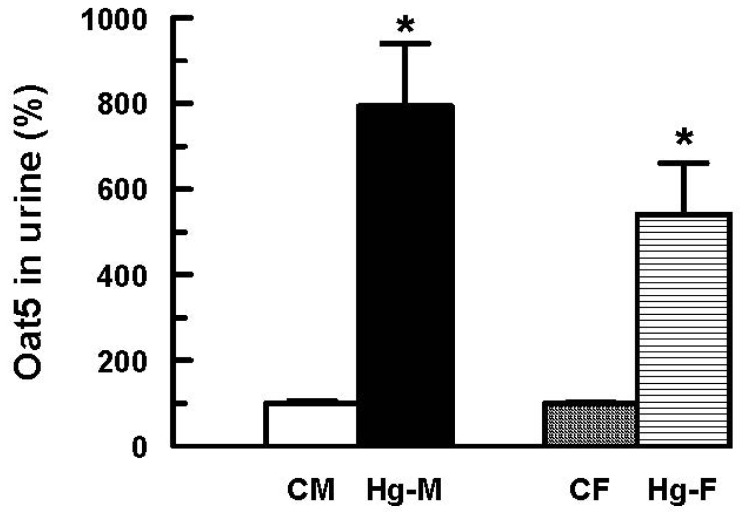
Effect of HgCl_2_ (4 mg/kg of body weight, i.p.) on urinary Oat5 excretion in male and female rats at 18 h after treatment. Each column represents the mean ± SEM from experiments carried out in four animals for each experimental group. * *p* < 0.05 *vs*. respective control. The mean of both control male and female levels were set as 100%. **CM**: Control Males; **Hg-M**: Hg-treated Males; **CF**: Control Females; **Hg-F**: Hg-treated Females.

We have recently postulated that the urinary excretion of Organic anion transporter 5 (Oat5) is an early biomarker of proximal tubule damage in ischemic acute kidney injury and in the nephropathy induced by HgCl_2_ [29,30]. Oat5 is located in the apical membranes of proximal tubule S3 segment where it functions as a dicarboxylate/organic anion transporter. Treatment of male rats with HgCl_2_ produced a significant increase in urinary excretion of Oat5 related to urinary creatinine levels (Figure 6). Following treatment with HgCl_2_, an increase of urinary Oat5 was observed in female rats compared with vehicle-treated females. However, this increase was moderately lower than that observed in HgCl_2_-treated male rats. The results obtained in this study also confirm the postulation of Oat5 as an early biomarker of proximal tubule damage in the nephrotoxicity induced by HgCl_2_.

Alkaline phosphatase activity and Oat5 abundance in urine were related to urinary creatinine concentrations to correct for variations in urine production as previously described for urinary transporters and enzymes [27–30]. Measurements of biomarker abundance alone are insufficient because normal physiological variations in water excretion can dilute or concentrate urinary proteins. Normalization on the basis of total protein amount is generally unsatisfactory because total protein excretion can vary broadly among various pathological states. Creatinine is excreted in the urine at relatively constant rates allowing it to be used to normalize urinary excretion of a particular protein.

**Figure 7:**
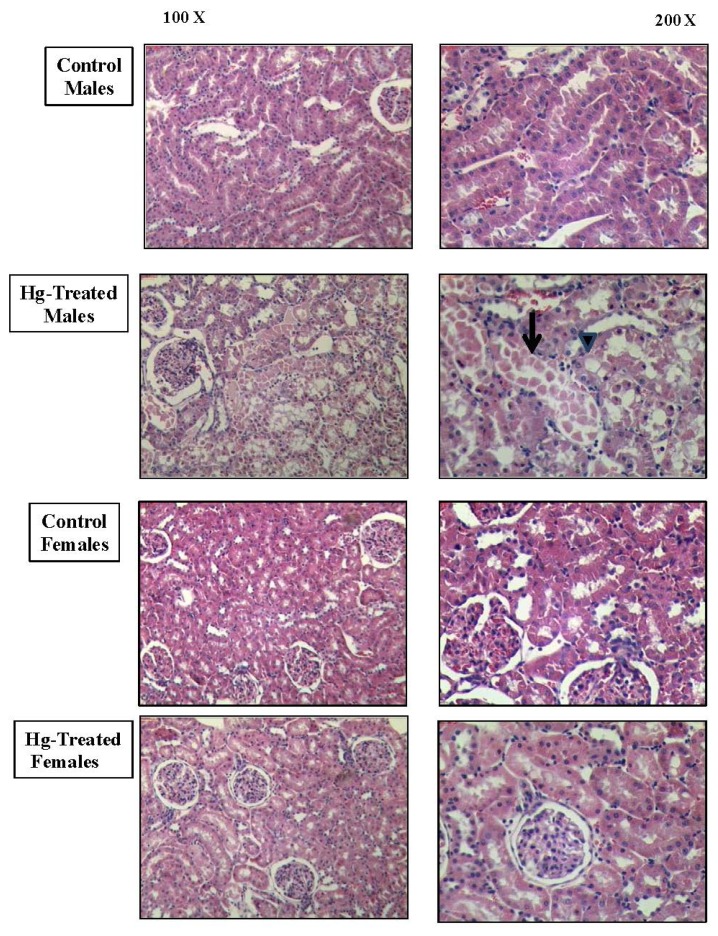
Representative micrographs of hematoxylin/eosin-stained sections of male and rat kidneys at 18 h following treatment with vehicle control or HgCl_2_ (4 mg/kg of body weight, i.p.). Magnification 100× and 200×.

Histopathological damage as assessed by hematoxylin/eosin-stained kidney sections revealed a marked difference in the pattern of renal injury between male and female rats after HgCl_2_ treatment (Figure 7). The male kidneys showed some vacuolated cells, disrupted brush border membranes, cellular detachment, disrupted tubular basement membranes and necrosis as previously described [29–32].

Di Giusto and Torres [30] have demonstrated similar histopathological damage in kidneys of male rats after the administration of HgCl_2_ (5 mg/kg b.w. subcutaneous, s.c.), which accounted for 2.37 μg/mL of mercury in urine samples. In Figure 7 it is possible to observe vacuolated cells (arrow head) and cellular detachment (arrow) in Hg-treated males. There were fewer microscopic changes suggestive of renal damage in the kidneys of Hg-treated females.

These data suggest that mercury-induced renal insufficiency is reduced in female rats as compared with males.

There is growing evidence that heavy metals, in general, and mercurial compounds, in particular, are toxic to humans. Large populations are currently exposed to low levels of mercury owing to the use of mercury (Hg) pesticides in agriculture or as components of batteries in fluorescent light bulbs [1–4]. All forms of mercury cause toxic effects in a number of tissues and organs [1,13]. Susceptibility to the injurious effects of mercury may be modified by a number of intracellular and extracellular factors. Physiological or pathological alterations in cellular function may also play important roles in modifying the susceptibility to mercury-induced renal injury [1,15].

In humans and other mammals, the kidneys are the primary targets of accumulation of mercuric ions after exposure to elemental or inorganic forms of mercury [15]. To understand the nephropathy induced by mercury and to find therapeutic regimens to treat this nephropathy, it is essential to understand the mechanisms involved in the uptake, intracellular binding, and cellular elimination of mercury in the target cells [15,31–33].

Hg^2+^ gains access to proximal tubular cells primarily via amino acid transporters in the luminal plasma membrane and the organic anion transporters 1 and 3 (Oat1 and Oat3) in the basolateral plasma membrane [15,18]. Bridges *et al.* [19] have recently demonstrated that Mrp2 plays an important role in the renal cellular elimination and secretion of Hg^2+^ in rats. Oat1 and Oat3 are involved in the organic anion transport into the renal cell at the basolateral membrane [34–36]. Oat1 is detected exclusively in the proximal tubules and Oat3 is localized in the proximal tubule, cortical and medullary thick ascending limb of Henle’s loop, connecting tubules, and cortical and medullary collecting ducts [37]. Both Oat1 and Oat3 support organic anion/α-ketoglutarate exchange [35,36].

Gender differences in the expression of Oat1 and Oat3 have been reported in mice and rats. The mRNA expression level of Oat1 and Oat3 in the kidney is higher in male rats than in female rats [21]. Gender differences in Oat1 and Oat3 expression have been confirmed at the protein level [22,23]. Oat1 and Oat3 protein levels in the renal cortex are higher in male rats than in female rats [22,23]. These differences are only observed in adult rats, not in prepubertal rats [23]. These differences are enhanced by androgens and inhibited by estrogens [23].

Considering that female rats have lower Oat1 and Oat3 renal expression than males, a lower nephrotoxicity induced by mercury was expected in female rats as compared to males.

In this study, a single injury dose of HgCl_2_ induced renal impairment both in male and female rats. However, female rats showed less renal impairment that males. The observed increase in kidney weight/body weight ratio seen in male and female rats following HgCl_2_ treatment was less in the female rats. The increase in the kidney weight observed in the HgCl_2_ group is presumably due to tissue water accumulation, as observed in other experimental models of acute renal failure [38–42].

In addition, other parameters of renal function perturbed by HgCl_2_ treatment were also less affected in female rats. Urine volume and creatinine clearance decreased in females to a lesser extent than in males. Oat5 urinary excretion increased after mercury treatment in both male and females, but to a lesser degree in the latter. Moreover, urinary AP activity after HgCl_2_ administration was increased in males and remained largely similar to control levels in females, whereas histological parameters were impaired in males and virtually unchanged in the female kidney following HgCl_2_ treatment. The lower Oat1 and Oat3 expression in kidney from females restricts Hg uptake into renal cells protecting them from this metal toxicity.

These gender differences in renal injury induced by mercury are striking and indicate that Oat1 and Oat3 are among the main transporters responsible for HgCl_2_-induced renal injury.

Our findings raise the possibility that pharmacological modulation of the expression and/or function of Oat1 and/or Oat3 might be an effective therapeutic strategy for reducing renal injury by mercury.

## 3. Experimental Section

### 3.1. Animals and Experimental Protocols

Male and female Wistar rats aged between 110–130 days were used throughout the study. All animals were allowed free access to a standard laboratory chow and tap water, and housed in a constant temperature and humidity environment with regular light cycles (12 h) during the experiment. All experiments were conducted according to National Institutes of Health (NIH), Guide for the Care and Use of Laboratory Animals [43].

Four experimental groups of four animals each were employed: Control Males, Control Females, Hg-treated Males and Hg-treated Females. Hg-treated animals were treated with a single injection (i.p) of HgCl_2_ at a nephrotoxic dose of 4 mg/kg body weight (*w*/*v* in 2 mL saline/kg) [15,20] and control groups received the vehicle alone (2 mL saline/kg). The studies were performed 18 h after the injection. During that period of time the animals were placed in metabolic cages in order to collect the urine. Urinary volume was determined by gravimetry.

Different sets of experimental animals were used for: biochemical determinations, histopathological studies and preparation of total plasma membranes for Western blotting studies.

On the day of the experiments the animals were anesthetized with sodium thiopental (70 mg/kg body weight, i.p.). The collection and processing of renal tissue samples was different depending on the type of study performed.

### 3.2. Biochemical Determinations

On the day of the experiments, blood samples were obtained by cardiac puncture and blood plasma was separated by centrifugation (1000*g* for 10 min). Urine samples were centrifuged at 1000*g* for 10 min to remove cell debris. The plasma samples were used for the determination of creatinine levels [Cr]p. The urine samples were used for analyses of Oat5 abundance, alkaline phosphatase (AP) activity and creatinine concentrations [Cr]o. Urine AP activity and creatinine levels, as well as plasma creatinine values were determined employing commercial kits (Alkaline Phosphatase optimized and Creatinine; respectively, by Wiener Laboratory, Rosario, Argentina). Creatinine clearance was calculated employing the following formula: [Cr]o × Urine Volume/[Cr]p. Urine Volume is expressed in mL/min/100 g b.w.

### 3.3. Preparation of Total Plasma Membranes from Kidneys

The preparation of total plasma membranes from controls and Hg-treated rats were performed according to the method described by Jensen and Berndt [44] as previously reported by our laboratory [22,24,33,38,45]. Animals were anesthetized and kidneys were removed surgically. Then, kidneys were decapsulated, washed in saline (9 g/L) at 4 °C and were then dried and weighed. The kidneys were cut off, minced and placed in a Dounce homogenizer containing 250 mM sucrose, 5 mM Tris-Hepes, pH 7.40, and 0.1 mg/mL phenylmethylsulphonyl fluoride (PMSF). After four gentle strokes with the loose fitting pestle, the suspension was homogenized further with a motor-driven Teflon pestle (600 rpm/5 strokes) and spun down for 15 min at 1200*g* and 4 °C. The supernatant was aspirated and spun for 15 min at 22,000*g* and 4 °C. The pellet that forms at the bottom of the tube is covered by a softer and beige layer called “fluffy”, which consists of crude plasma membranes. This layer was resuspended in about 500 μL of buffered sucrose.

Aliquots of the membranes were stored immediately at −80 °C for 2 weeks. Each preparation represented renal tissues from four animals. Protein quantification of samples was performed using the method of Sedmak and Grossberg [46].

### 3.4. Electrophoresis and Immunoblotting

Total plasma membranes (18 μg of protein/lane) and urine (10μL/lane) samples were boiled for 3 min in the presence of 1% 2-mercaptoethanol/2% sodium dodecyl sulphate (SDS). Proteins were separated through 8.5% SDS-polyacrylamide gel electrophoresis (SDS-PAGE), and then electroblotted to a pure nitrocellulose membrane (NC membrane) (Trans-Blot^®^ Transfer Medium, Bio Rad Laboratories, Hercules, CA, USA). To verify equal protein loading and transfer between lanes, Ponceau Red was used as previously described [38,45]. The NC membranes were incubated with 5% non-fat dry milk in phosphate-buffer saline containing 0.1% Tween 20 (PBST) (80 mM Na_2_HPO_4_, 20 mM NaH_2_PO_4_, 100 mM NaCl, 0.1% Tween 20, pH 7.50) for 1 h. After being rinsed with PBST, the NC membranes were incubated overnight at 4 °C with a commercial rabbit polyclonal antibody against rat Oat1 (1.25 μg/μL) or with a non-commercial rabbit polyclonal antibody against rat Oat3 (at a dilution of 1/1000) or with a non-commercial rabbit polyclonal antibody against rat Oat5 (at a dilution of 1 *vs*. 800). The specificity of Oat3 and Oat5 antibodies has been described elsewhere [37,47]. The NC membranes were incubated for 1 h with a peroxidase-coupled goat anti-rabbit IgG (Zymed^®^, Invitrogen, Carlsbad, CA, USA) after further washing with PBST. Blots were processed for detection using a commercial kit (ECL Plus Western Blotting Detection Reagents; Amersham, Buckinghamshire, UK). Kaleidoscope Prestained Standards of molecular mass were employed (Bio Rad Laboratories, Hercules, CA, USA).

A densitometric quantification of the Western blotting signal intensity of membranes was performed using the Gel-Pro Analyzer (Media Cybernetics, Silver Spring, MD, USA) software. For densitometry of Oat1 and Oat3 immunoblots, samples from kidneys of control females rats were run on each gel with male control kidneys. The abundance of Oat1 and Oat3 in the samples from the female animals was calculated as percentage of the mean male value for that gel.

For densitometry of Oat5 immunoblots, samples from kidneys of Hg-treated rats were run on each gel with the corresponding control kidneys for each sex. The abundance of urine Oat5 in Hg-treated animals was calculated as percentage of the mean control value of the same sex for that gel.

### 3.5. Histopathological Studies

Histopathology of kidneys was performed after fixing in 10% neutral buffered formaldehyde solution for 4 h and embedding in paraffin; 4-μm-thick sections were processed for routine staining with hematoxylin-eosin.

### 3.6. Materials

Chemicals were purchased from Sigma (St. Louis, MO, USA) and were analytical grade pure. The polyclonal antibody against Oat1 was purchased from Alpha Diagnostic International (San Antonio, TX, USA). The polyclonal antibodies against Oat3 and Oat5 were kindly given by Prof. H. Endou and Prof. N. Anzai (Department of Pharmacology and Toxicology, Kyorin University School of Medicine, Tokyo, Japan).

### 3.7. Statistical Analysis

Statistical analysis was performed using the unpaired Student’s *t*-test. When variances were not homogeneous a Welch’s correction was employed. *p* < 0.05 was considered statistically significant. The values are expressed as the means ± standard error (S.E.M.). For these analyses, GraphPad software was used.

## 4. Conclusion

The results indicate that the lower Oat1 and Oat3 expression in kidney from female rats restricts Hg uptake into renal cells, protecting them from this metal toxicity. These gender differences in renal injury induced by mercury are striking and also indicate that Oat1 and Oat3 are among the main transporters responsible for HgCl_2_-induced renal injury. Current work in our laboratory aims to evaluate if the pharmacological modulation of the expression and/or function of Oat1 and/or Oat3 are effective therapeutic strategies for reducing renal injury induced by mercury.

## Figures and Tables

**Table 1:** Effect of HgCl_2_ (4 mg/kg of body weight (b.w.), intraperitoneal (i.p.)) on the ratio of kidney weight to body weight, urinary volume, creatinine clearance and in the urinary activity of alkaline phosphatase (AP) in male and female rats 18 h after treatment. Results are expressed as mean ± SEM. The experiments were carried out in four animals for each experimental group.

	CM	Hg-M	CF	Hg-F
Renal/Body weigth ratio × 10^−3^	6.49 ± 0.11 (100 ± 2)	8.27 ± 0.31 * (127 ± 5 *)	6.81 ± 0.22 (100 ± 3)	8.12 ± 0.14 *(119 ± 2 *)
Urinary Volume × 10^−3^ (mL/min/100 g b.w.)	2.38 ± 0.52 (100 ± 22)	0.88 ± 0.18 * (37 ± 7 *)	3.31 ± 0.60 (100 ± 18)	2.22 ± 0.44 * (67 ± 13 *)
Creatinine clearance × 10^−3^ (mL/min/100 g b.w.)	421 ± 80(100 ± 19)	35 ± 3 * (8 ± 1 *)	349 ± 60 (100 ± 17)	85 ± 24 * (24 ± 7 *)
Urinary AP Activity (mUI/mg creatinine)	165 ± 26(100 ± 16)	5511 ± 2167 * (3340 ± 1313 *)	230 ± 24 (100 ± 10)	578 ± 169 (251 ± 73)

* *p* < 0.05 *vs*. respective control. Percentages are indicated in brackets and the mean of both control male and female levels were set as 100%.

**CM**: Control Males, **Hg-M**: Hg-treated Males; **CF**: Control Females; **Hg-F**: Hg-treated Females.
